# Changes in cognitive performance following repeated exposure to a hand-touch learning task across breed clades of domestic dogs (*Canis familiaris*)

**DOI:** 10.1007/s10071-026-02044-6

**Published:** 2026-01-16

**Authors:** Amin Azadian, Alexandra Protopopova

**Affiliations:** Animal Welfare Program, Faculty of Land and Food Systems, University of British Columbia, Vancouver, BC Canada

## Abstract

Selective breeding for specialized functional roles has resulted in consistent differences among domestic dog breeds in a range of cognitive capacities, as demonstrated by standardized cognitive assessments. What remains uncertain is whether these breed differences extend beyond initial task performance to influence how dogs adjust their behaviour or improve their performance when they are re-tested on the same tasks. In the present study, we used a structured hand-touch learning task to examine changes in Discrimination, Reversal learning, and resistance to Extinction performances across two testing sessions in 105 dogs representing five breed clades. Statistical analyses accounted for demographic variables, reward responsiveness, impulsivity, and training history. Dogs showed limited changes in learning performance across sessions, with statistically significant improvement observed only in Reversal learning from Test 1 to Test 2. Most breed-clade differences in average performance found during the initial test were no longer evident at Test 2. There was also limited evidence that breed clades differed in the extent of their performance change over time. Taken together, these results suggest that initial performance differences across breed clades likely reflect breed-specific cognitive profiles that shape how dogs engage with novel learning tasks. Following re-exposure to the task, average performance tends to improve and potentially mask the initial between-clade differences, although the magnitude of improvement appears broadly similar across breed clades. These findings highlight the importance of considering both initial performance and prior experience when interpreting breed differences in learning and cognitive capacities.

**Supplementary Information:**

The online version contains supplementary material available at 10.1007/s10071-026-02044-6.

## **Introduction**

A growing body of research suggests that centuries of selective breeding for specialized working tasks have likely shaped the cognitive profile of purebred domestic dogs (*Canis familiaris*), aligning each breed’s cognitive capacities with the demands associated with their historical functions (Azadian and Protopopova 2024; Dutrow et al. 2022; Junttila et al. 2022; MacLean et al. 2019; Pongrácz and Dobos 2024). As a result, dogs of different breeds may show variations in how they approach and perform in cognitive tasks (Azadian and Protopopova 2024).

However, much of this work in canine cognition research has focused on breeds’ task performance at a single time point, leaving it unclear whether these breed-specific cognitive profiles also reflect differential capacity to improve performance with experience.

Human studies showed that the extent of performance change associated with stable cognitive abilities may vary across populations and depend on factors such as the specific cognitive domain assessed (Breit et al. 2021, 2024). Thus, change in cognitive performance may not be uniform even within a species, raising the question of whether a similar pattern exists in domestic dogs.

Most existing longitudinal assessments in domestic dogs has focused on developmental changes (Bray et al. 2021; Lazarowski et al. 2023) or age-related trajectories (Chapagain et al. 2020; González-Martínez et al. 2013; Piotti et al. 2018; Wallis et al. 2020), leaving open the question of how selective breeding might affect the extent of performance changes over time.

Certain dog populations or breeds may benefit more from training and repeated task exposure, whereas others may show little change despite practices (e.g., differential effect of training experience on the executive function of working and non-working dogs; Foraita et al. 2021, 2023). Moreover, these performance changes may not be uniform across cognitive capacities, as certain types of learning performances may show substantial change with experience, whereas others may remain largely unchanged and repeatable (e.g., Bognár et al. 2024).

In the present study, we examined differences in average (mean-level) cognitive performance among five breed clades using a structured hand-touch learning task administered on two separate time points (30 days interval). The task assessed several cognitive capacities sequentially within the same task structure—Discrimination, Reversal, and Extinction learning—which together capture how animals acquire, update, and apply information under changing contingencies.

Discrimination learning reflects the ability to differentiate and respond to relevant environmental stimuli, forming the basis of information acquisition and decision-making (Wallis et al. 2016). Reversal learning builds on this capacity by assessing the ability to modify previously learned associations when reward contingencies change, thereby supporting adaptive learning and change in performance (Van Bourg et al. 2021). Resistance to extinction, in turn, captures behavioural persistence when reinforcement is withdrawn, revealing how individuals maintain a learned stimulus-response association following reward loss (Hall 2017).

Dogs’ frustration-like behaviours were also measured as a non-cognitive trait that may modulate performance across repeated trials within a session, since sensitivity to reward loss, frustration, or task-related arousal may negatively influence performance (e.g., increasing errors after non-reward) (Azadian and Protopopova 2024; McPeake et al. 2019).

Examining changes in performance is important as these changes may provide important context for interpreting the initially documented differences between breeds. Thus, the study aimed to address three complementary research questions: (1) Does the average (mean-level) performance of each breed clade improve from Test 1 to Test 2 (within-breed performance change), (2) do between-breed clade performance differences found during the initial assessment (Test 1; if any) persist in Test 2, and (3) do breed clades differ in the average extent of change associated with their performance scores across tests (between-breed difference in performance change).

We hypothesized that breed clades would, on average, show improvement in Discrimination and Reversal learning performances upon re-exposure to the task, accompanied by reduced resistance to Extinction and frustration-like behavioural expressions following experience with the task. We also expected that the between-breed clade differences observed at Test 1 would be attenuated at Test 2, as task experience may differentially benefit lower-performing compared to high-performing clades and reduce the performance gaps initially observed between breed clades. We further hypothesized that the magnitude of performance change would differ across breed clades, reflecting variation in the degree to which different cognitive capacities have been shaped by selective breeding.

## Methods

### Ethical statement

All procedures adhered to the UBC guidelines and regulations, and the research protocol received approval from the UBC Human Research Ethics Board (H22-00509) and the Animal Care Committee (A22-0025). Informed consent was obtained from all dog owners for their participation in the study, during which their performance was recorded on a video.

### Subjects

A total of 151 dogs from five breed clades were recruited for an initial study, exploring cross-sectional variations in learning and behavioural performances across and within five breed clades (data published in Azadian and Protopopova 2024). Breed clades comprised of the UK Rural (*n* = 28), Asian Spitz (Ancient dog breeds) (*n* = 31), Retriever (*n* = 32), New World (represented only by the German Shepherd dogs– *n* = 27), and European Mastiff (*n* = 33) clades.

Out of the 111 dogs that successfully finished Test 1, four dogs (two from the New World, one from the Asian Spitz, and one from the European Mastiff clades) failed to successfully finish the task in their second testing occasion (Test 2), while two more dogs (one from the UK Rural and one from the Retriever clades) were also excluded as their owners opted out of the study after their participation in Test 1. A total of 105 dogs were included in the final study population for the current study. The dogs’ age range (in years) was 0.5–11.58 (4.16 ± 0.188; Std. dev: 2.74); out of the 105 dogs, 57 (54.28%) were female and 68 (64.8%) were spayed/neutered.

### Behavioural questionnaire

Before participating in the initial study, owners of the recruited dogs were requested to fill out a questionnaire consisting of Canine Reward Responsiveness Scale (Gerencsér et al. 2018) and Dog Impulsivity Assessment Scale (Wright et al. 2011) along with questions focusing on dogs’ demographic information and training background. Questions on dogs’ training background focused on two aspects: (1) whether the dog has had training for Agility and/or Rally/Obedience (given the similarity in the structure of these trainings to the hand-touch learning task; see below) and have actively participated in these activities or not (data was pulled as a categorical variable: Yes or No, labeling dogs either as a “sport” or “non-sport” dog); (2) whether the dog has been trained to perform a hand-touch behavioural command (i.e., nose-touching the palm for a reward) before participating in the study.

### Requirements for the experimental task

The hand-touch task was conducted virtually and recorded via Zoom^®^ Video Communications, Inc. (“Zoom” version of 5.3.0.). Owners performed the task with their dogs while a researcher (AA) guided them throughout the experimental session.

Owners were advised to perform the task in a quiet area/room, having enough space for the dog to move a few steps around and in front of them. Behavioural sessions were scheduled at different times during the day, depending on the owners’ availability, with each session taking approximately 20–75 min (depending on the dog’s performance).

Throughout the experimental session, owners were instructed to wear sunglasses (to prevent any potential gazing effect on dogs’ performance), remain silent (except when they were required to speak; e.g., verbally reinforcing the touching behaviour of the dog by saying “YES” after a correct response – see below), and avoid providing any physical cues that could possibly influence the dog’s performance (e.g., engage them to respond to the presented hand). Furthermore, owners were asked to use their dog’s most favorite food treat during the experimental sessions, ensuring that the treat was either small in size or could be cut and divided it into smaller pieces/cubes, facilitating the food consumption. Treats should have either be placed in the right and left pockets of a training vest or in a treat pouch (placed behind the owner’s waist) throughout the experimental sessions.

### Learning task procedure and measures

The hand-touch learning task comprised of four different phases, including Acquisition (or pre-training), Discrimination learning (DL), Reversal learning (RL), and Extinction (EXT) (Azadian and Protopopova 2024). To successfully finish all phases of the task (from Acquisition to EXT), dogs had a maximum participation opportunity of three days for Test 1 and two days for Test 2. All re-scheduled dogs (i.e., those that were unable to finish all task phases on a given experimental day), regardless of which phase they failed, were required to start the task from the beginning (the Acquisition phase) on the next experimental testing day.

For the Acquisition (pre-training) phase, dogs were trained to do a nose-touch on the owner’s palm. The goal of this phase was to familiarize the dog with the task procedure and to ensure they were motivated to engage in more cognitively demanding sessions involving the touching behaviour. Thus, dogs’ performance was not recorded or analyzed throughout this phase. The owner was asked to present only one of their hands at a time (starting with their dominant hand), and alternating the presented hand (e.g., from right to left, and vice versa) trial-by-trial, with the dog having a maximum of 10 s to respond (i.e., nose-touch) after the hand’s presentation. The touching response was then immediately reinforced with a food treat by the owner, using the same hand presented to the dog.

To meet the Acquisition criteria, the dog was required to make four consecutive touching responses (minimum of two touches on each hand), each within three seconds after the owner presented the hand. Each Acquisition session comprised of a maximum of 20 trials, with a total of 60 trials (maximum of three Acquisition sessions) allowed per experimental testing day for the dog to meet the Acquisition criteria.

In the case of having three consecutive trials with response omission (i.e., no response from the dog within the 10-second limit after the hand’s presentation; labelled as a “No-response” trial), the Acquisition session was terminated, and another session started after one minute. If the dog was unable to meet the Acquisition criterion over three Acquisition sessions (within each experimental testing day) (i.e., either reached the maximum 60 trials or had three consecutive sessions terminated due to response omission), they were re-scheduled to participate on a different day (within a maximum of seven days after their previous attempt).

The Acquisition phase was divided into two parts. Dogs that met the performance criteria in the initial part completed an additional Acquisition session (using the same criteria) before proceeding to the next phase of the task (i.e., the Discrimination learning [DL] phase). This additional session was intended to minimize any potential side bias toward one of the owner’s hands before starting the DL phase.

The DL phase was started immediately after the dog successfully met the Acquisition criteria for the second time. The goal of the DL phase was to evaluate dogs’ ability to differentiate between two identical stimuli (i.e., hands). Therefore, owners were asked to present both of their hands at the same time, while only reinforcing the touching response on their dominant hand. The DL phase consisted of multiple sessions (10 trials each – maximum of 6 sessions per each experimental testing day, with one-minute inter-session breaks), and the performance criterion for the dog was to touch the target (dominant) hand in 8 out of 10 given trials (80% accuracy) within a session.

The next phase of the task (i.e., Reversal learning [RL]) started after a two-minute time break, once the dogs met the DL performance criteria. The goal of the RL phase was to evaluate the dogs’ ability of modifying their behaviour following changes in the reinforcement contingency. The RL phase procedure remained the same as the DL phase, whereas the target hand was switched to the owner’s non-dominant hand. Each dog was required to perform a total six RL sessions (with one-minute inter-session breaks), regardless of the outcome, to standardize the number of sessions performed across dogs and overcome learning confounds (e.g., reduce the variance in the number of food rewards eaten) prior to the last phase of the task.

Similar to the Acquisition phase, three consecutive trials with response omission resulted in the termination of the given DL or RL session. If three consecutive sessions were terminated due to response omission, the experiment was discontinued for that day, and the dog was rescheduled for another day—unless they had already reached the maximum number of allowed attempts.

After a two-minute break, the last phase of the task (i.e., Extinction [EXT]) was conducted to evaluate dogs’ perseverance (i.e., continuing a previously reinforced behaviour while the reinforcement is withheld). The EXT phase was conducted as a single, continuous session, where the dog’s response on either of the owner’s hands was no longer reinforced. The EXT session continued until the dog demonstrated three consecutive trials with response omission (considered as the EXT breakpoint).

**Fig. 1:**
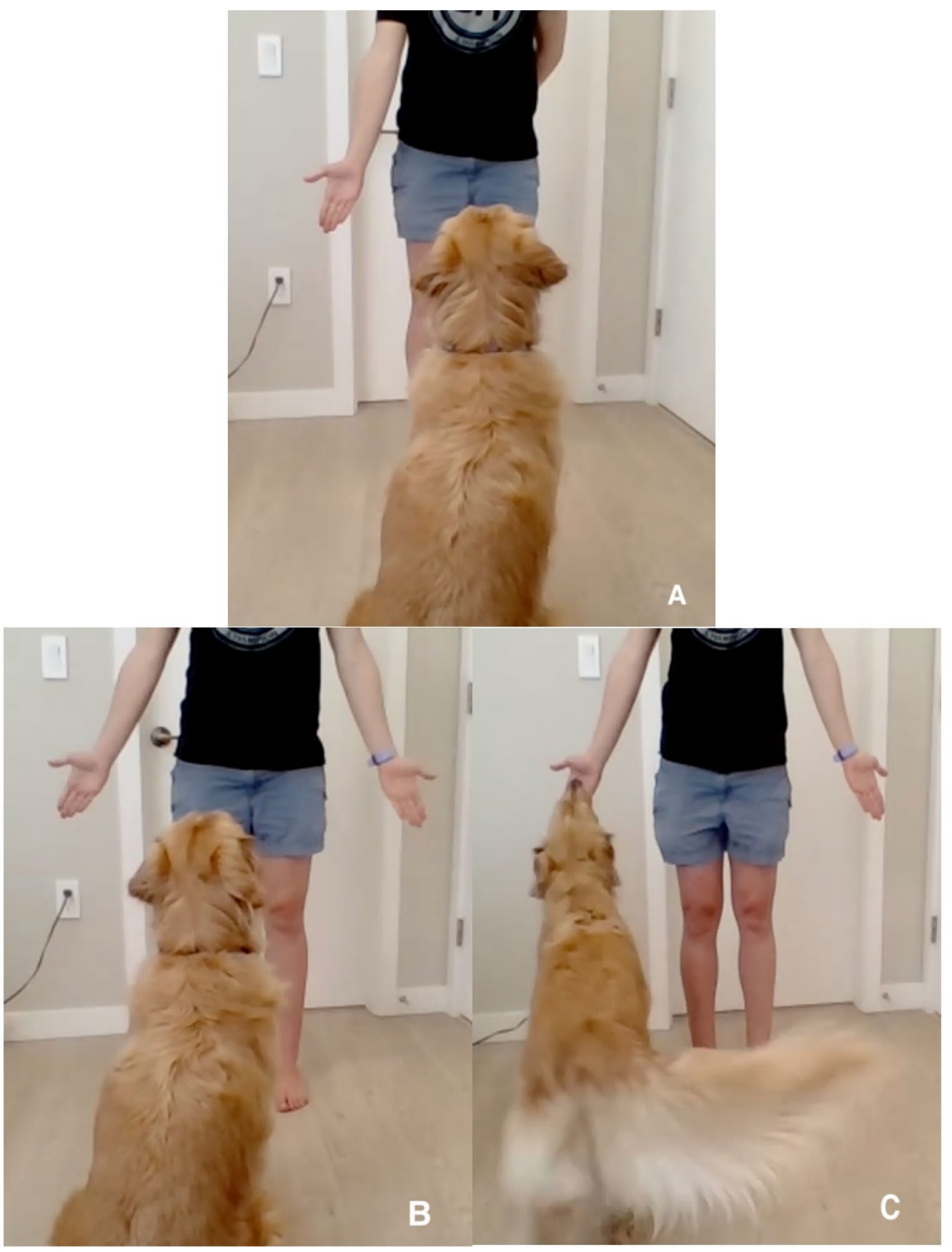
Illustrations of the experimental task setup and procedure. **A**) Pre-training (Acquisition) phase - the owner presents one hand at a time; **B** and **C**) Main task phases - the owner presents both hands simultaneously

All dogs that successfully completed Test 1 were the invited to re-take the same, identical task (i.e., Test 2) approximately 30 to 35 days later (depending on the owner’s availability). For a detailed procedure and special considerations of the task used in the current study see Azadian and Protopopova (2024) and Fig. 1.

Frustration-like behaviours exhibited by the dogs were assessed and quantified through video coding of task performances. The goal was to evaluate how individual dogs cope with learning challenges and how their behavioural responses change upon re-exposure to the same task. The percentage of trials in which frustration-like behaviours were observed—including vocalisations (e.g., barking and whining), jumping on the owner, pawing at the owner, and nipping—was recorded (see Azadian and Protopopova 2024).

Inter-rater reliability for frustration-like behaviours was assessed via re-coding 15% of the videos by a second researcher blinded to the study objectives. Reliability was evaluated using Intraclass Correlation Coefficient (ICC) analysis. Results showed an average measure of 0.966 (95% CI [0.931, 0.983]; *p* < 0.001) for the Discrimination learning (DL), 0.957 (95% CI [0.914, 0.979]; *p* < 0.001) for the Reversal learning (RL), and 0.940 (95% CI [0.878, 0.970]; *p* < 0.001) for the Extinction (EXT) phases.

### Statistical analysis

A total of 13 variables were recorded based on dogs’ performance in the task (see Azadian and Protopopova 2024). All variables were standardised (z-scored) across both testing sessions combined. This approach ensured that both time points were expressed on an identical metric and without confounding effects of re-scaling or re-centring at each session separately.

A Principal Component Analysis (PCA) was conducted on the standardized data of Test 1 to determine the number of extracted components and within-component variable structures, along with loading values and direction of loadings associated with each variable. Despite the exclusion of some dogs, the variable structure and loading directions were consistent with the analysis performed in the initial study (Azadian and Protopopova 2024) (see Table 1 for the loadings of individual variables).

**Table 1:** Results of the rotated component matrix conducted on the data of test 1. Loading values for individual variables along with communalities are presented

Standardized variables of Test 1	Component				Communalities
	1 (Difficulty in Discrimination learning performance)	2 (Difficulty in Reversal learning performance)	3 (Emotionality)	4 (Resistance to Extinction/Perseverance)	
% of sessions terminated in Discrimination	**0.832**	0.127	−0.095	−0.108	0.728
Sessions took until meeting the Discrimination criteria	**0.849**	−0.037	0.102	0.053	0.736
% of correct trials in Discrimination	**−0.871**	0.177	0.066	0.012	0.794
% of No-response trials in Discrimination	**0.841**	0.212	0.052	−0.217	0.802
% of sessions terminated in Reversal learning	0.018	**0.917**	−0.011	−0.185	0.876
% of correct trials in Reversal learning	0.002	**−0.883**	−0.054	0.034	0.785
% of No-response trials in Reversal learning	0.06	**0.921**	0.051	−0.269	0.927
% of No-response trials in Extinction	0.198	0.366	0.03	**−0.717**	0.689
Total Extinction responses	−0.001	−0.119	0.032	**0.906**	0.836
Number of times the dog switched between the two hands	−0.058	−0.068	0.164	**0.778**	0.64
% of trials with emotional responses in Discrimination	0.1	−0.07	**0.905**	−0.058	0.837
% of trials with emotional responses in Reversal learning	−0.086	0.13	**0.926**	0.061	0.885
% of trials with emotional responses in Extinction	−0.022	0.038	**0.856**	0.19	0.77

Extraction Method: Principal Component Analysis

Rotation Method: Varimax with Kaiser Normalization.^a^

a. Rotation converged in 4 iterations

Four components (explaining 79.2% of the total variance for Test 1) were extracted from Test 1’s standardized data and labelled based on the variables and their loading directions as “difficulty in Discrimination learning” and “difficulty in Reversal learning”, “Perseverance”, and “Emotionality”. Higher values (i.e., higher component scores) for the first two components (associated with the DL and RL phases) described experiencing more performance difficulty (or being less engaged with the task), while higher values for the Perseverance and Emotionality components described higher levels of resistance to EXT and frustration-like behaviours expressed by the dog, respectively.

Given the variables measured on each testing occasion were identical, we decided to adopt factor loadings extracted from Test 1’s PCA to calculate and create a set of scores for the standardized variables of Test 2. Specifically, each dog’s score on a given component in Test 2 was calculated by multiplying the standardized individual values associated each variable from Test 2 by its corresponding component loading value extracted from Test 1’s PCA solution and summing across the set of variables corresponding to that component.

This method has been previously used in psychometric and longitudinal data analysis research in other scientific disciplines (e.g., in human nutrition for the study of changes in the dietary pattern over time – see Mishra et al. 2006; Northstone and Emmett 2008; Prevost et al. 1997; see also Bognár et al. 2024 – using item loadings from baseline PCA to calculate component scores for the subsequent testing occasion performed by dogs). Using this approach (i.e., projecting new data onto an existing PCA solution) allowed a direct comparison of component scores across testing occasions conducted at different time points, ensuring that the same underlying component structure is maintained for both datasets (using the same measurement scale), while also reducing potential biases introduced by independently derived factor solutions.

To assess the first two research questions, Generalized Linear Mixed Models (GLMM) with a repeated measure design were conducted. Normal distribution with an identity link function was used for target distribution since, although the variables were slightly right skewed, their distributions were approximately symmetric and continuous, and residual diagnostics indicated that a Gaussian model provide an adequate fit while allowing effect estimates to be interpreted on the original scale.

The variable “Time” was included as the repeated measure (within-subject) variable, while the learning/behaviour components served as the target variable (in separate models). For the fixed effects, “Time” was included along with the collected demographic factors (breed clade, sex, and age [in years]), sport training experience, reward responsiveness scores (Food responsiveness [FR] and Ball/Toy responsiveness [BTR]), the overall Dog Impulsivity Assessment Scale (DIAS) score, and dogs’ prior experience of the hand-touch command.

To examine whether the average performance score changed from Test 1 to Test 2 within each breed clade and whether the initially documented between-breed differences (in Test 1; if any) persisted or potentially changed in direction following re-exposure to the task, an interaction effect between the variables “Breed clade” and “Time” was included in the model. This interaction term also directly tests whether the change in learning and behavioural performance from Test 1 to Test 2 differs between breed clades while appropriately accounting for the repeated-measures error structure.

However, to complement and aid interpretation of the mixed-model results, we additionally conducted a confirmatory follow-up analysis in which generalized linear models (GLMs) were fitted to the calculated difference (Δ) in learning and behavioural component scores between Test 1 and Test 2 (Test 1  − Test 2), with breed clade included as a fixed predictor.

For the assessment of fixed effects and coefficients, the robust estimation was used to handle violations of model assumptions (using HC3 robust estimator - Hayes and Cai 2007). Sequential Sidak was used as the *p*-value adjustment method for multiple comparisons of estimated marginal means.

Since the initial PCA revealed that performance measures associated with each task phases (i.e., Discrimination, Reversal, and Extinction) loaded on separate components with minimal cross-loadings, these measures appeared to capture largely distinct sources of variance. Therefore, performance scores from earlier phases performed by dogs were not included as covariates in the models ran for subsequent phases, as including them could introduce multicollinearity.

Statistical analyses were performed using the analytical software package SPSS 29.0 (SPSS, Chicago, IL, USA). Alpha level was set at 0.05 and *p*-values less than this level were denoted as statistically significant. Data were presented as Mean ± Std. error of the mean (SEM) unless otherwise specified. Tables of the fixed effects are presented in the supplementary material file. Only results associated with the average (mean-level) change in performance of dogs and breed clades over time are reported in the manuscript.

## Results

### Descriptive information

Information on the distribution of breeds, age, sex, along with dogs’ ball/toy responsiveness (BTR), food responsiveness (FR), and overall impulsivity (DIAS) scores were provided in Table 2. Out of the 105 dogs included in the final data, 21 were from the breeds belonging to the UK Rural clade, 19 to the Asian Spitz clade, 22 to the Retriever clade, 20 to the New World (GSDs), and 23 to the European Mastiff clade. The BTR score of dogs ranged from 1.142 to 4.928 (3.061 ± 0.104; Std. dev: 1.073), the FR score ranged from 1.3 to 5.0 (3.388 ± 0.084; Std. dev: 0.866), and the overall DIAS score ranged from 0.311 to 0.820 (0.515 ± 0.008; Std. dev: 0.091).

**Table 2:** Descriptive information on the participating dogs successfully passed both testing occasions (*N* = 105). Information on age, sex, breed clade, along with average food and ball/toy responsiveness and impulsivity scores were provided. Ball/toy responsiveness is written as “BTR”, food responsiveness as “FR”, and the overall impulsivity score as “DIAS” in the table

Breed Clades	Age range in years (mean ± Std. Deviation)	Sexual status	Breeds	Number of dogs	Total	Reward responsiveness and Impulsivity (mean ± Std. Deviation)
UK rural clade	0.66–11.0 (3.68 ± 2.776)	Neutered Male: 2 Intact Male: 6 Spayed Female: 12 Intact Female: 1	Border Collie Corgi Australian Shepherd Shetland Sheepdog	4	21	BTR (3.543 ± 0.852) FR (3.252 ± 0.844) DIAS (0.525 ± 0.11)
				5		
				11		
				1		
Retriever clade	0.75–11.58 (4.5 ± 2.815)	Neutered Male: 10 Intact Male: 3 Spayed Female: 6 Intact Female: 3	Nova Scotia Duck Tolling Retriever Labrador Retriever Golden Retriever	7 8 7	22	BTR (3.094 ± 1.156) FR (3.868 ± 0.688) DIAS (0.491 ± 0.073)
New World clade	0.75–9.0 (3.769 ± 2.173)	Neutered Male: 4 Intact Male: 4 Spayed Female: 7 Intact Female: 5	German Shepherd Dog	20	20	BTR (3.541 ± 0.926) FR (2.8 ± 0.782) DIAS (0.485 ± 0.068)
European Mastiff clade	1.166–11.416 (4.067 ± 2.869)	Neutered Male: 8 Intact Male: 7 Spayed Female: 5 Intact Female: 3	Rhodesian Ridgeback	4	23	BTR (2.856 ± 1.027) FR (3.613 ± 0.716) DIAS (0.538 ± 0.09)
			Great Dane	2		
			Cane Corso	2		
			Boerboel	1		
			Staffordshire Bull Terrier	6		
			English Bull Terrier	4		
			Boston Terrier	2		
			Bulldog	2		
Asian Spitz clade	0.5–11.25 (4.841 ± 3.082)	Neutered Male: 4 Intact Male: 3 Spayed Female: 9 Intact Female: 3	Siberian Husky	15	19	BTR (2.232 ± 0.889) FR (3.331 ± 0.981) DIAS (0.535 ± 0.103)
			Alaskan Malamute	1		
			Shiba Inu	3		

A total of 12 dogs (three UK Rural dogs, five Asian Spitz dogs, and four Retriever dogs) used more than one day to successfully meet the performance criteria and finish all task phases for Test 1 (two of the five Asian Spitz dogs used the maximum three-day limit for Test 1; Azadian and Protopopova 2024), and five dogs used the maximum two-day limit to finish the task for Test 2 (two Asian Spitz dogs, one European Mastiff dog, one Retriever dog, and one New World dog).

### Change in discrimination learning (DL) performance

**Fig. 2:**
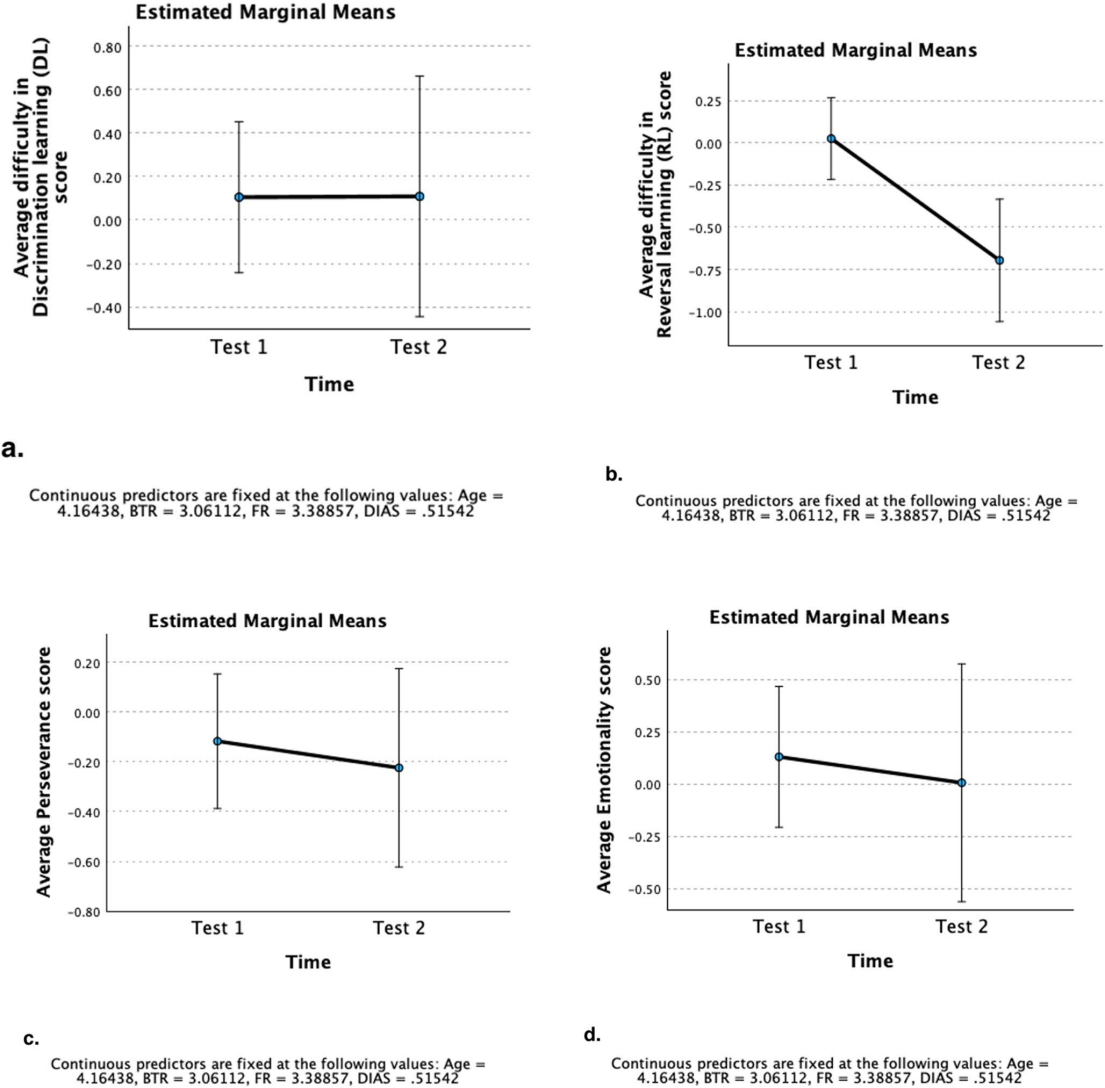
Estimated Marginal Means of component scores: **a**) “difficulty in Discrimination learning” score, **b**) “difficulty in Reversal learning” score, **c**) “Perseverance” score, and **d**) “Emotionality” across Test 1 and Test 2. For Discrimination and Reversal learning, higher scores describe more difficulty in performance, while for Perseverance and Emotionality, higher scores describe higher levels of behavioural exhibition. Error bars are 95% Confidence Interval (CI) and values are the estimated marginal means

**Fig. 3:**
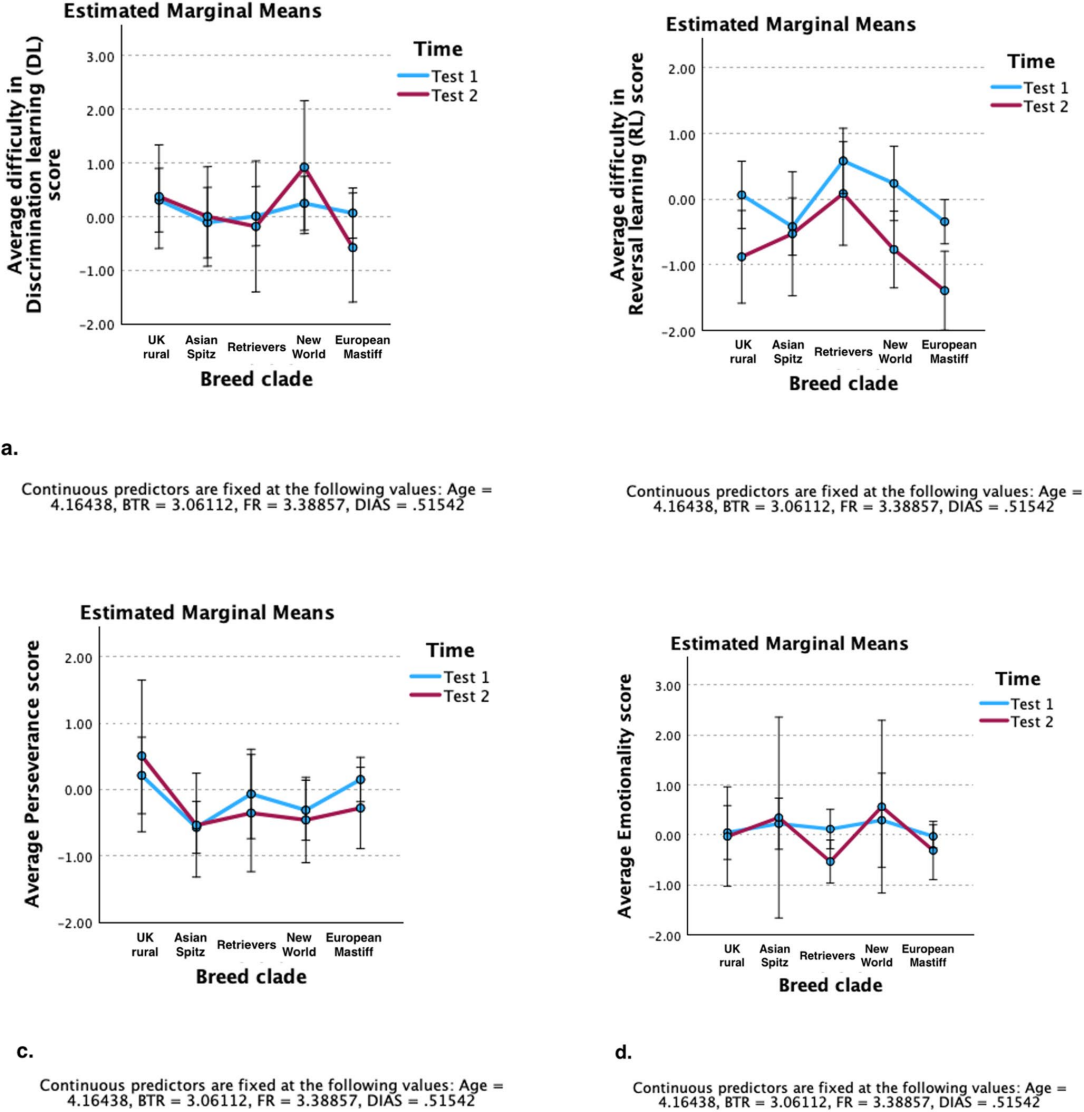
Estimated marginal means of component scores: **a**) “difficulty in Discrimination learning”, **b**) “difficulty in Reversal learning”, **c**) “Perseverance” score, and d) “Emotionality” by breed clade across Test 1 and Test 2. For Discrimination and Reversal learning, higher scores describe more difficulty in performance, while for Perseverance and Emotionality, higher scores describe higher levels of behavioural exhibition. Error bars are 95% Confidence Interval (CI) and values are the estimated marginal means

Result of the mixed model analysis (Corrected model: *F*(16, 122) = 1.569, *p* = 0.087 - Table S1 in the supplementary material file) showed that the effect of “Time” (*F*(1, 80) = 0.00, *p* = 0.989) on the “difficulty in DL” score was not statistically significant (Fig. 2-a). Pairwise between-test comparisons of the estimated marginal means within each breed clade (Fig. 3-a) also indicated that the average “difficulty in DL” score of breed clades remained relatively consistent across Test 1 and Test 2 (Table S2). None of the between-breed clade differences in the average “difficulty in DL” score within Test 1 and Test 2 reached statistical significance (Table S3). Pairwise contrasts on the average change in “difficulty in DL” score (Test 1 – Test 2) also showed no significant difference between the studied breed clades (Figure S1-a and Table S4).

### Change in reversal learning (RL) performance

The mixed model analysis (Corrected model: *F*(16, 126) = 3.764, *p* < 0.001 - Table S5) showed that the average “difficulty in RL” score of dogs significantly changed across the two testing occasions (*F*(1, 72) = 16.829, *p* < 0.001), with dogs generally showing a significant improvement in their average performance score (experiencing less difficulty) from Test 1 to Test 2 (contrast estimate [Test 1 – Test 2]: 0.721 ± 0.176, *t* = 4.102, Adj. *p* < 0.001) (Fig. 2-b).

Pairwise between-test comparison of estimated marginal means (Fig. 3-b) revealed that several breed clades showed significant decreases—albeit to varying degrees—in their average “difficulty in RL” scores from Test 1 to Test 2. Significant improvements were observed in the average score of UK Rural (contrast estimate [Test 1 – Test 2]: 0.942 ± 0.327, *t* = 2.885, Adj. *p* = 0.004), New World (contrast estimate [Test 1 – Test 2]: 1.006 ± 0.367, *t* = 2.743, Adj. *p* = 0.007), and European Mastiff clades (contrast estimate [Test 1 – Test 2]: 1.051 ± 0.334, *t* = 3.150, Adj. *p* = 0.002). However, the average “difficulty in RL” scores of the Asian Spitz and Retriever clades did not significantly change from Test 1 to Test 2 (Table 3).

**Table 3:** Pairwise comparisons of the estimated marginal means for “difficulty in reversal learning” score across test 1 and test 2 within the studied breed clades. Breed clades that are bolded had a statistically significant change in their mean-level performance

Breed clade	Contrast Estimate (Test 1 - Test 2)	Std. Error	t	df	Adj. Sig.	95% Confidence Interval	
						Lower	Upper
UK Rural*	**0.942**	**0.327**	**2.885**	**193**	**0.004**	**0.298**	**1.587**
Asian Spitz	0.109	0.489	0.224	25	0.825	−0.898	1.117
Retrievers	0.497	0.426	1.168	62	0.247	−0.354	1.348
New World*	**1.006**	**0.367**	**2.743**	**153**	**0.007**	**0.282**	**1.730**
European Mastiff*	**1.051**	**0.334**	**3.150**	**136**	**0.002**	**0.391**	**1.710**

The sequential Sidak adjusted significance level is 0.05

Confidence interval bounds are approximate

Pairwise contrasts between the studied breed clades revealed that the Retriever and European Mastiff clades significantly differed in their average “difficulty in RL” score within both Test 1 and Test 2 (contrast estimate for Test 1 [Retriever – European Mastiff]: 0.926 ± 0.292, *t* = 3.174, Adj. *p* = 0.017; contrast estimate for Test 2 [Retriever – European Mastiff]: 1.479 ± 0.485, *t* = 3.048, Adj. *p* = 0.033). However, the significant mean-level difference initially documented between the Retriever and the Asian Spitz clade in Test 1 (contrast estimate [Asian Spitz – Retriever]: −1.001 ± 0.352, *t* = −2.843, Adj. *p* = 0.044) did not reach statistical significance in Test 2 (contrast estimate [Asian Spitz – Retriever]: −0.613 ± 0.592, *t* = −1.035, Adj. *p* = 0.772) (Table 4). Furthermore, pairwise contrasts examining the average change in “difficulty in RL” scores (Test 1 – Test 2) revealed no significant differences among the studied breed clades (Figure S1-b and Table S6).

**Table 4:** Pairwise comparisons of the estimated marginal means for “difficulty in reversal learning” score between the studied breed clades within test 1 and test 2. Breed comparisons that are bolded had a statistically significant difference in their mean-level performance

Time	Breed clade Pairwise Contrasts	Contrast Estimate	Std. Error	t	df	Adj. Sig.	95% Confidence Interval	
							Lower	Upper
Test 1	UK Rural – Asian Spitz	0.482	0.309	1.558	193	0.538	−0.34	1.305
	UK Rural - Retrievers	−0.519	0.353	−1.469	193	0.539	−1.434	0.397
	UK Rural – New World	−0.175	0.334	−0.524	193	0.841	−0.927	0.577
	UK Rural – European Mastiff	0.407	0.306	1.33	186	0.559	−0.363	1.178
	Asian Spitz - Retrievers*	**−1.001**	**0.352**	**−2.843**	**193**	**0.044**	**−1.985**	**−0.016**
	Asian Spitz - New World	−0.657	0.332	−1.98	193	0.332	−1.572	0.258
	Asian Spitz - European Mastiff	−0.075	0.264	−0.283	193	0.841	−0.618	0.468
	Retrievers - New World	0.344	0.389	0.883	193	0.76	−0.594	1.282
	Retrievers – European Mastiff*	**0.926**	**0.292**	**3.174**	**193**	**0.017**	**0.1**	**1.752**
	New World - European Mastiff	0.582	0.34	1.714	193	0.476	−0.339	1.504
Test 2	UK Rural – Asian Spitz	−0.351	0.566	−0.619	36	0.902	−1.768	1.067
	UK Rural - Retrievers	−0.964	0.533	−1.808	63	0.506	−2.49	0.563
	UK Rural – New World	−0.111	0.426	−0.261	192	0.902	−1.019	0.797
	UK Rural – European Mastiff	0.516	0.414	1.244	153	0.702	−0.562	1.594
	Asian Spitz - Retrievers	−0.613	0.592	−1.035	30	0.772	−2.182	0.957
	Asian Spitz - New World	0.239	0.525	0.456	41	0.902	−1.002	1.481
	Asian Spitz - European Mastiff	0.867	0.554	1.564	25	0.567	−0.714	2.447
	Retrievers - New World	0.852	0.505	1.688	71	0.536	−0.558	2.263
	Retrievers – European Mastiff*	**1.479**	**0.485**	**3.048**	**64**	**0.033**	**0.072**	**2.887**
	New World - European Mastiff	0.627	0.37	1.696	193	0.536	−0.393	1.647

The sequential Sidak adjusted significance level is 0.05

Confidence interval bounds are approximate

### Change in perseverance (resistance to extinction)

Results of the mixed model analysis (Corrected model: *F*(16, 120) = 2.303, *p* = 0.006 – Table S7) showed that the average “Perseverance” score of dogs did not significantly differ between Test 1 and Test 2 (*F*(1, 51) = 0.486, *p* = 0.489), reflecting a relatively consistent average level of resistance to Extinction in dogs across the two tests (contrast estimate [Test 1 – Test 2]: 0.107 ± 0.154, *t* = 0.697, Adj. *p* = 0.489) (Fig. 2-c).

None of the studied breed clades showed statistically significant differences in their average “Perseverance” score across the two testing occasions (Fig. 3-c and Table S8). Nevertheless, pairwise contrasts of the average “Perseverance” score between breed clades showed a significant mean-level difference between the Asian Spitz and European Mastiff clades in Test 1 (contrast estimate [Asian Spitz – European Mastiff] = − 0.721 ± 0.224, *t* = − 3.221, adj. *p* = 0.015). This difference, however, did not reach statistical significance in Test 2 (contrast estimate [Asian Spitz – European Mastiff] = − 0.258 ± 0.475, *t* = − 0.544, adj. *p* = 0.995) (Table 5). Furthermore, the average change in “Perseverance” scores (Test 1 – Test 2) were comparable between the studied breed clades (Fig. 1-c and Table S9).

**Table 5:** Pairwise comparisons of the estimated marginal means for “Perseverance” score between the studied breed clades within test 1 and test 2. Breed comparisons that are bolded had a statistically significant difference in their mean-level performance

Time	Breed clade Pairwise Contrasts	Contrast Estimate	Std. Error	t	df	Adj. Sig.	95% Confidence Interval	
							Lower	Upper
Test 1	UK Rural – Asian Spitz	0.783	0.309	2.531	193	0.104	−0.082	1.648
	UK Rural - Retrievers	0.279	0.456	0.612	45	0.944	−0.889	1.447
	UK Rural – New World	0.523	0.278	1.881	193	0.398	−0.243	1.289
	UK Rural – European Mastiff	0.062	0.328	0.188	107	0.944	−0.645	0.768
	Asian Spitz - Retrievers	−0.504	0.362	−1.393	47	0.673	−1.497	0.489
	Asian Spitz - New World	−0.26	0.266	−0.981	193	0.863	−0.949	0.428
	Asian Spitz - European Mastiff*	**−0.721**	**0.224**	**−3.221**	**193**	**0.015**	**−1.356**	**−0.087**
	Retrievers - New World	0.244	0.433	0.563	52	0.944	−0.843	1.33
	Retrievers – European Mastiff	−0.217	0.331	−0.658	71	0.944	−1.062	0.627
	New World - European Mastiff	−0.461	0.286	−1.61	143	0.556	−1.24	0.318
Test 2	UK Rural – Asian Spitz	1.043	0.602	1.733	26	0.63	−0.795	2.88
	UK Rural - Retrievers	0.86	0.73	1.179	15	0.875	−1.414	3.134
	UK Rural – New World	0.964	0.557	1.73	22	0.63	−0.76	2.689
	UK Rural – European Mastiff	0.784	0.609	1.287	18	0.855	−1.094	2.662
	Asian Spitz - Retrievers	−0.183	0.589	−0.31	54	0.997	−1.706	1.34
	Asian Spitz - New World	−0.079	0.488	−0.161	141	0.997	−1.247	1.089
	Asian Spitz - European Mastiff	−0.258	0.475	−0.544	119	0.995	−1.529	1.012
	Retrievers - New World	0.104	0.549	0.189	55	0.997	−1.253	1.46
	Retrievers – European Mastiff	−0.076	0.523	−0.145	65	0.997	−1.334	1.182
	New World - European Mastiff	−0.18	0.445	−0.404	193	0.997	−1.334	0.974

The sequential Sidak adjusted significance level is 0.05

Confidence interval bounds are approximate

### Change in emotionality

The model predicting dogs’ “Emotionality” (Corrected model: (16, 41) = 2.669, *p* = 0.006; Table S10) showed that the average score of dogs did not significantly change from Test 1 to Test 2 (*F*(1, 13) = 0.605, *p* = 0.451) (contrast estimate [Test 1 – Test 2]: 0.124 ± 0.159, *t* = 0.778, Adj. *p* = 0.451) (Fig. 2-d).

Pairwise between-test contrasts indicated that, among the studied breed clades, only the Retriever clade showed a significant decrease in average “Emotionality” score from Test 1 to Test 2 (contrast estimate [Test 1 – Test 2]: 0.651 ± 0.147, *t* = 4.440, Adj. *p* < 0.001) (Table 6). In contrast, no statistically significant between-test changes were observed for the remaining breed clades.

**Table 6:** Pairwise comparisons of the estimated marginal means for “Emotionality” score across test 1 and test 2 within the studied breed clades. Breed clades that are bolded had a statistically significant change in their mean-level performance

Breed clade	Contrast Estimate (Test 1 - Test 2)	Std. Error	t	df	Adj. Sig.	95% Confidence Interval	
						Lower	Upper
UK Rural	0.079	0.296	0.267	83	0.790	−0.509	0.668
Asian Spitz	−0.123	0.505	−0.243	2	0.829	−2.081	1.836
Retrievers*	**0.651**	**0.147**	**4.440**	**193**	**< 0.001**	**0.362**	**0.941**
New World	−0.270	0.454	−0.595	18	0.559	−1.223	0.683
European Mastiff	0.282	0.255	1.106	193	0.270	−0.221	0.785

The sequential Sidak adjusted significance level is 0.05

Confidence interval bounds are approximate

Additionally, pairwise contrasts between breed clades revealed no significant differences in average “Emotionality” scores within either Test 1 or Test 2 (Table S11). Comparisons of the magnitude of change in average “Emotionality” scores (Test 1 – Test 2) across breed clades similarly showed no significant differences (Fig. 1-d and Table S12).

## Discussion

The current study examined whether breed-specific cognitive profiles are associated with variation in dogs’ capacity to change or improve their performance with experience. Specifically, we investigated whether all breed clades show performance changes following re-exposure to the task, whether such changes attenuate or modify previously documented performance differences across breed clades, and whether changes in performance are domain-specific, affecting only particular learning capacities.

Findings revealed that among the assessed learning and behavioural components, dogs only showed significant improvements in their average Reversal learning performance. This finding is consistent with previous research (albeit in different animal species), showing improved performance levels following additional experiences (through repeated exposure) with Reversal learning tasks. Examples can be found in serial Reversal learning experiments, where performance improved across successive reversals as subjects developed a learning set. Previous research found this learning sets to facilitate more efficient switching between the two stimuli, resulting in fewer trials to reach a pre-defined performance criteria (e.g., in bumblebees – Strang and Sherry 2014; in bats – Chidambaram et al. 2024; in rats – Watson et al. 2006).

The significant between-test improvement in average Reversal learning performance, however, was not uniform across the studied breed clades, with only three (UK Rural, New World, and European Mastiff) of the five clades showing significant changes in their performance difficulty scores. Similar patterns have been observed in other taxa. For example, Reichert et al. (2020) investigated how contextual and individual factors influenced task performance in wild mixed-species bird flocks during an initial Discrimination learning task followed by two Reversal learning tests, with the second Reversal mirroring the original Discrimination rule (i.e., using the same discriminative stimulus). Results showed that great tits displayed less change in their performance throughout the sessions compared to blue tits. Similarly, our findings suggest that experience-related improvements in Reversal learning are not equally detectable across all dog breed clades, even under the same protocol.

The absence of a statistically significant performance change in the other two breed clades, however, does not necessarily imply that their performance remained unchanged. Factors such as greater within–breed clade (individual) variability may have made improvements more difficult to detect statistically. Thus, while only some clades showed clear evidence of reduced Reversal difficulty at the group level, the overall pattern is still consistent with the hypothesis that repeated exposure can possibly enhance Reversal learning performance, with the strength and statistical visibility of this effect potentially varying across breed clades.

Despite the improvement observed in Reversal learning performance, dogs’ average Discrimination performance did not significantly change across the two tests. This pattern is consistent with the evidence that Discrimination and Reversal learning may rely on partly independent cognitive mechanisms (Dalley et al. 2004; Schoenbaum et al. 2002). However, the absence of improvement in Discrimination learning contrasts with several studies that report improvement in Discrimination performance following repeated exposure. For example, Wilson et al. (2023) reported that dogs required fewer trials to reach the Discrimination criterion during their second exposure to a Judgement Bias Task (JBT), and Krahn et al. (2024) similarly reported faster Discrimination criterion attainment during a second JBT session.

The discrepancy between these findings and those of the current study may stem from methodological differences (JBT vs. Hand-touch task). Variations in task structure (e.g., different type of stimuli), sample composition, or the overall difficulty of reaching performance criterion may all influence whether improvements in Discrimination learning emerge upon repeated testing.

Alternatively, many dogs may have already performed at a relatively moderate (near-optimal) level during their initial task exposure (resulting in ceiling effects), leaving limited room for improvement. In Test 1, dogs required an average of 2.22 ± 0.14 sessions to reach the Discrimination performance criterion and achieved an average of 75.9% ± 1.53 correct choices. In Test 2, they required 2.61 ± 0.15 sessions on average, with an average of 69.3% ± 1.87 correct choices. These values suggest that, compared to Reversal learning—where the initial performance difficulty was notably higher (e.g., 55.7% ± 2.24 correct choices in Reversal of Test 1 compared to 69.71% ± 1.63 in Test 2)—the initial Discrimination performance may have provided a relatively smaller and limited room for improvement across tests.

Another potential reason for the differential effect of “Time” on Discrimination versus Reversal learning may be attributed to the sequential structure of the task. Although Test 2 followed the same procedural sequence as Test 1, the function of the stimuli (i.e., hands) had changed by the time dogs re-encountered them in Test 2. In Test 1, the owner’s dominant hand served as the discriminative stimulus in the Discrimination learning phase, and later, it was subsequently associated with non-reward (extinction stimulus or S-delta) during the Reversal and Extinction phases. Consequently, when dogs began Test 2, the owner’s dominant hand no longer carried a purely reward-based history but instead had a combination of reward and extinction histories and experiences. This mixed reinforcement history may have influenced how dogs responded when the same stimuli was re-introduced in the second test, further limiting the degree of improvement observed in Discrimination learning phase.

One of the potential reasons behind this mixed reinforcement history effect could be proactive interference. Proactive interference occurs when previously learned contingencies conflict with those that are newly presented (due to their similarity), potentially impairing the acquisition of new associations and disrupting performance in subsequent learning tasks (see Morand-Ferron et al. 2022). Although this accumulated learning history may not have directly affected performance at later phases of Test 1, it could have created additional challenges when dogs were re-exposed to a similar sequence of contingencies in Test 2. In particular, the absence of a significant mean-level change—especially for Discrimination learning—may reflect a memory-based interference effect: dogs may have perceived the Discrimination phase of Test 2 not as a re-instatement of the original Discrimination task but rather as another Reversal relative to the contingencies they last experienced in Reversal learning phase of Test 1. This could increase cognitive load and impair the acquisition of the updated stimulus–response association, as previously learned rules compete with new ones (Crossley et al. 2019; Lewis and Kamil 2006; Morand-Ferron et al. 2022).

Although proactive interference is primarily associated with Reversal rather than the Discrimination learning performance (Morand-Ferron et al. 2022), if the Discrimination phase in Test 2 was perceived as a Reversal learning phase (similar to a serial Reversal task), proactive interference could be a potential explanation for the lack of between-test difference found in average Discrimination learning performance.

Since the task structure resembles a simplified serial Reversal learning procedure (Discrimination to Reversal and then Extinction in Test 1, followed by Discrimination and Reversal in Test 2), the significant improvement in the average Reversal performance could also be attributed to the development of a Reversal set that facilitates switching after multiple sessions. In other words, while proactive interference may have led to difficulty in re-learning the original Discrimination association, the structure of the task and the accumulated reinforcement experience across the two tests may have facilitated the acquisition of a general “Reversal rule”, enabling dogs to update their responses more efficiently when contingencies shifted again during the Reversal phase of Test 2 (i.e., progressive improvement – see Chidambaram et al. 2024; Erdsack et al. 2022). Thus, dogs’ improvement in Reversal learning appears to be consistent with the development of a broader behavioural strategy that supports adjusting behavioural responses to series of sequential Reversals, despite potential memory-based interference that may happen during the Discrimination learning phase upon re-exposure to the task.

Future research should examine whether methodological adjustments, such as introducing longer intervals between the learning phases or separating Discrimination, Reversal, and Extinction into distinct tasks rather than embedding them within a single multi-phase task sequence, would provide a clearer estimate of performance change associated with learning and cognitive capacities.

Similar to the Discrimination learning performance, the average resistance to Extinction (i.e., Perseverance) exhibited by the studied breed clades did not significantly differ across the two time points. However, previous findings on changes in Perseverance over time have been inconsistent across animal species. For example, Mahoney et al. (2015) found that a second exposure to the Extinction produced a more rapid decline in responding rate of mine-detection rats. In contrast, studies in dogs, such as Lazzaroni et al. (2020), found Perseverance (labelled as behavioural persistence) to remain consistent over time and unaffected by context or prior experiences.

Interpreting these discrepancies is, however, difficult, given that Perseverance has been defined and operationalized in different ways across studies, depending on the task used. In some contexts (including the present study), perseverative responding refers to continued responding toward a stimulus even after the point that responses no longer produce reinforcement (Eagle and Baunez 2010). Other work has quantified Perseverance as the amount of time an individual persists in attempting to solve an impossible task (Gould et al. 2022; Khan et al. 2023). As a result, the type of Perseverance captured in our hand-touch extinction phase may not be generalizable to other tasks unless those tasks closely resemble the current procedure in structure and performance demands.

In the context of Extinction learning, the degree to which a previously reinforced behaviour resists Extinction is proportional to the strength of its reinforcement history and inversely proportional to the intensity of the disruptor applied (e.g., the extinction procedure itself; Hall 2017). Furthermore, studies using serial reversal learning tasks have shown that perseverative errors typically decrease as individuals receive or experience more testing trials (Erdsack et al. 2022; Strang and Sherry 2014). It is therefore possible that a single re-exposure to the Extinction phase in Test 2 did not serve as a sufficiently strong disruptor to reduce perseverative responding in dogs. The Discrimination and Reversal phases preceding the second Extinction phase might have also re-instated or strengthened the previously reinforced behaviour, thereby maintaining the resistance to Extinction levels in Test 2.

Since the hand-touch task involves multiple shifts in contingencies across phases—exposing the dog to repeated loss of expected rewards, a known trigger for disappointment- or frustration-like responses (see McPeake et al. 2019, 2021)—we also examined whether Emotionality changed across the two tests. Among the studied breed clades, only Retrievers showed a significant reduction in their average Emotionality score from Test 1 to Test 2. Notably, Retrievers were among the two breed clades that did not demonstrate significant improvements in their average Reversal performance across the two tests. Therefore, the documented decrease in Retriever’s frustration-like behaviours might reflect their breed-specific behavioural response toward increasing familiarity with the task or decreasing the amount of uncertainty experienced regarding task contingencies following re-exposure to the task, rather than improved performance efficiency per se.

We also examined whether the within-breed changes in average performance across the two tests altered the initially observed between-breed differences (Test 1). Pairwise comparisons between breed clades revealed a mixed pattern. For the Reversal learning performance, difference in average performance between Retrievers and European Mastiffs persisted across both tests, indicating a consistent between-breed variation over time. In contrast, the significant difference initially observed between Asian Spitz and Retrievers was no longer present in Test 2.

Udell et al. (2014) examined point-following performance across dog breeds that underwent differential selective pressures on predatory motor patterns and found that breed-specific performance differences could diminish when initially lower-performing breeds gained additional practice experience with the task. Although point-following is not directly comparable to the hand-touch learning task used here, both tasks require dogs to respond to human hand cues. A similar pattern may help explain the disappearance of the Reversal learning difference between the Asian Spitz and Retriever clades. Retrievers showed a modest, albeit non-significant, reduction in their average Reversal difficulty score from Test 1 to Test 2, whereas Asian Spitz dogs—one of the highest-performing clades—displayed minimal mean-level change. Thus, improvement in the initially lower-performing clade (Retrievers), combined with limited change in the higher-performing clade (Asian Spitz), may have obscured the between-breed difference that was initially present at Test 1.

We also examined whether breed clades differed in the extent to which their performance changed across the two time points (Test 1 – Test 2). Although several breed clades showed significant improvements in their average Reversal learning performance, the average magnitude of change in performance scores did not significantly differ between the studied breed clades. This pattern indicates that, despite variations in initial performances, experience-related changes occurred broadly across breed clades. Thus, selective breeding and variation in cognitive capacities may have exerted stronger effects on the initial task performances rather than the degree to which performance changes by additional task experience. Once dogs have gained experience with the task, their ability to change performance may therefore reflect a more general learning capacity shared across breed clades, rather than capacities that diverge strongly along breed-specific cognitive profiles.

## Limitations of the study and future directions

Several limitations should be acknowledged for the present study. The structure of the task involved various phases and sessions being performed consecutively within the same experimental session. Thus, fatigue or boredom resulting from the repetitive nature of the task in earlier phases may have further influenced performance in later phases. These factors further highlight the limitations of using a combined task design, suggesting that future studies may benefit from employing separate, independent tasks to assess changes in various learning capacities and cognitive domains more accurately.

Another key limitation of the present study lies in the inability to control for potential owner-mediated practice or reinforcement of the learned behaviour during the interval between the two testing sessions. Although the performance associated with the additional sessions conducted for the re-scheduled dogs were incorporated in the calculations and analysis (see Azadian and Protopopova 2024), it remains uncertain whether some owners continued practising or reinforcing the hand-touch behaviour at home. Such unsupervised training could have significantly influenced dogs’ subsequent task performance, particularly among individuals that initially showed greater difficulty in the first test (which may lead to significant improvements in performance upon re-exposure to the task in Test 2). Longitudinal designs with more controlled re-exposure conditions would further clarify how prior experience and spontaneous practice contribute to improvements in cognitive performance over time.

While the use of PCA-derived component scores enhanced interpretability and reduced dimensionality, these scores may obscure important features of the dogs’ performances, such as variation at the trial-level or specific behavioural/error patterns, limiting the granularity of the interpretation. Another limitation concerns the operationalization of complex components such as “perseverance” and “emotionality,” which, although derived from variable reduction techniques, may still reflect overlapping constructs that could not be generalizable and defined as a latent behavioural or cognitive component.

Having only two testing occasions to evaluate change in the average learning and behavioural performances may create constrains, especially given the small sample size associated with each breed clade. Future studies should include more dogs along with additional testing sessions to follow each dog’s individual learning pattern and to confirm whether the Reversal learning improvement seen in the current study truly reflects a broader ability to adjust learned behaviours upon changes in environmental contingencies.

Although the findings of the current study align with previous research on Reversal learning improvement over time, their generalizability to other Reversal learning paradigms may be limited, given the task-specific features of the hand-touch learning task. In particular, dogs typically have extensive and highly consistent reinforcement histories with their owners’ hands, which may enhance salience, reduce ambiguity, and facilitate rapid learning relative to reversal tasks that employ less socially meaningful stimuli, such as containers or buttons. Consequently, performance in the hand-touch learning task may reflect a combination of reversal-learning ability and stimulus-specific familiarity that may not fully generalize to other experimental contexts.

Nevertheless, the hand-touch task captures core components of Reversal learning that are shared across paradigms, including the inhibition of a previously reinforced response, and the re-allocation of behaviour following negative feedback. To the extent that these cognitive demands are common across Reversal learning tasks, the observed patterns of performance change and experience-related improvements are likely to generalize at the level of underlying learning processes, even if absolute performance levels differ across paradigms or experimental tasks.

Generalizability may also be influenced by the sequential structure of the task, as dogs experienced multiple contingency shifts across sessions. Such cumulative exposure may alter behavioural allocation strategies over time as Reversal experiences are accumulated through multiple sessions. Thus, this structure may limit direct comparability with simpler reversal paradigms, although it may somehow mirror real-world learning contexts in which animals encounter repeated and evolving contingencies.

Lastly, the sample composition using breed clades (with some breeds being unintentionally over-represented) may limit generalizability of findings to specific breeds. Future research with more diverse samples and counterbalanced designs may help further validate and expand upon these findings.

## Conclusion

Results of the present study revealed a significant improvement in average Reversal learning performance of dogs following re-exposure to the hand-touch task, whereas no comparable changes were observed for Discrimination, Perseverance, or Emotionality. Improvement was evident only in some of the studied breed clades, and there were no differences among breed clades in the magnitude of performance change associated with any of the learning or behavioural component. Together, these patterns suggest that the benefits of additional experience may be specific to certain cognitive capacities (most notably reversal learning), while remaining broadly similar in magnitude across breed clades.

Breed clades may initially differ in their average performance, reflecting underlying cognitive profiles and differences in how each breed approach novel learning tasks. However, experience gained during the first task exposure may alter subsequent performance in ways that reduce or mask these initial between-breed clade differences. Factors inherent to the task structure may also have contributed to the limited detectable changes in performance associated with some components. Since the discriminative stimuli carried both reinforcement and extinction histories across testing sessions, processes such as proactive interference or the resurgence of previously reinforced responses may have constrained further reductions in Discrimination difficulty, Perseverance, or Emotionality, despite increased task familiarity.

## Supplementary Information

Below is the link to the electronic supplementary material.

Supplementary Material 1

## Data Availability

The dataset for the current study is available at doi:10.5683/SP3/8MC0 × 2.
