# Supplementary material for: Changes in cognitive performance following repeated exposure to a hand-touch learning task across breed clades of domestic dogs (Canis familiaris)
Source: Anim Cogn. 2026 Jan 16;29(1):20. doi: 10.1007/s10071-026-02044-6 (PMC12860876; doi:10.1007/s10071-026-02044-6)
Supplement: Supplementary file 1 — Supplementary Material 1 [file 10071_2026_2044_MOESM1_ESM.docx]

**Table S1.** **Fixed effects table from the mixed model analysis predicting “difficulty in Discrimination learning” score (N=105).** Ball/toy responsiveness is written as “BTR”, Food responsiveness is written as “FR”, and overall Impulsivity score written as “DIAS”. Statistically significant variables are bolded.

| Fixed Effects^a^ | F | df1 | df2 | Sig. |
| --- | --- | --- | --- | --- |
| Corrected Model | 1.569 | 16 | 122 | 0.087 |
| Breed clade | 1.218 | 4 | 78 | 0.310 |
| Age | 2.137 | 1 | 168 | 0.146 |
| Sex | 0.709 | 1 | 68 | 0.403 |
| Sport training | 0.218 | 1 | 63 | 0.643 |
| Hand-touch experience | 0.798 | 1 | 35 | 0.378 |
| BTR | 0.007 | 1 | 82 | 0.935 |
| **FR*** | **8.600** | **1** | **99** | **0.004** |
| DIAS | 0.799 | 1 | 56 | 0.375 |
| Time | 0.000 | 1 | 80 | 0.989 |
| Breed clade * Time | 0.711 | 4 | 75 | 0.587 |
| Probability distribution: Normal | | |  |  |
| Link function: Identity^a^ | |  |  |  |

**Table S2.** **Pairwise comparisons of** **the Estimated Marginal Means for “difficulty in Discrimination learning” score across Test 1 and Test 2 within the studied breed clades.** Breed clades that are bolded had a statistically significant difference in their mean-level performance.

| Breed Group | Contrast Estimate (Test 1 - Test 2) | Std. Error | t | df | Adj. Sig. | 95% Confidence Interval | |
| --- | --- | --- | --- | --- | --- | --- | --- |
|  |  |  |  |  |  | Lower | Upper |
| UK Rural | -0.065 | 0.548 | -0.119 | 119 | 0.905 | -1.151 | 1.02 |
|  |  |  |  |  |  |  |  |
| Asian Spitz | -0.114 | 0.525 | -0.218 | 182 | 0.828 | -1.15 | 0.921 |
|  |  |  |  |  |  |  |  |
| Retrievers | 0.192 | 0.629 | 0.305 | 43 | 0.762 | -1.077 | 1.461 |
|  |  |  |  |  |  |  |  |
| New World | -0.672 | 0.65 | -1.034 | 53 | 0.306 | -1.977 | 0.632 |
|  |  |  |  |  |  |  |  |
| European Mastiff | 0.642 | 0.497 | 1.292 | 97 | 0.199 | -0.344 | 1.627 |
| The sequential Sidak adjusted significance level is 0.05.  Confidence interval bounds are approximate. | | | | | | | |

**Table S3. Pairwise comparisons of the Estimated Marginal Means for “difficulty in Discrimination learning” score between the studied breed clades within Test 1 and Test 2.** Breed comparisons that are bolded had a statistically significant difference in their mean-level performance.

| Time | Breed clade Pairwise Contrasts | Contrast Estimate | Std. Error | t | df | Adj. Sig. | 95% Confidence Interval | |
| --- | --- | --- | --- | --- | --- | --- | --- | --- |
|  |  |  |  |  |  |  | Lower | Upper |
| Test 1 | UK Rural – Asian Spitz | 0.419 | 0.416 | 1.005 | 193 | 0.978 | -0.761 | 1.598 |
|  | UK Rural - Retrievers | 0.298 | 0.377 | 0.79 | 193 | 0.989 | -0.742 | 1.337 |
|  | UK Rural – New World | 0.06 | 0.289 | 0.207 | 193 | 0.99 | -0.618 | 0.738 |
|  | UK Rural – European Mastiff | 0.241 | 0.342 | 0.703 | 193 | 0.99 | -0.687 | 1.168 |
|  | Asian Spitz - Retrievers | -0.121 | 0.37 | -0.327 | 193 | 0.99 | -1.027 | 0.785 |
|  | Asian Spitz - New World | -0.359 | 0.407 | -0.882 | 193 | 0.986 | -1.496 | 0.778 |
|  | Asian Spitz - European Mastiff | -0.178 | 0.339 | -0.525 | 193 | 0.99 | -1.056 | 0.701 |
|  | Retrievers - New World | -0.238 | 0.336 | -0.707 | 193 | 0.99 | -1.149 | 0.674 |
|  | Retrievers – European Mastiff | -0.057 | 0.301 | -0.19 | 193 | 0.99 | -0.757 | 0.643 |
|  | New World - European Mastiff | 0.181 | 0.312 | 0.58 | 193 | 0.99 | -0.639 | 1.001 |
| Test 2 | UK Rural – Asian Spitz | 0.37 | 0.625 | 0.592 | 149 | 0.952 | -1.191 | 1.93 |
|  | UK Rural - Retrievers | 0.555 | 0.739 | 0.751 | 55 | 0.952 | -1.411 | 2.522 |
|  | UK Rural – New World | -0.547 | 0.741 | -0.738 | 64 | 0.952 | -2.501 | 1.407 |
|  | UK Rural – European Mastiff | 0.948 | 0.664 | 1.428 | 87 | 0.785 | -0.935 | 2.83 |
|  | Asian Spitz - Retrievers | 0.186 | 0.783 | 0.237 | 49 | 0.952 | -1.587 | 1.959 |
|  | Asian Spitz - New World | -0.917 | 0.744 | -1.232 | 71 | 0.828 | -2.972 | 1.139 |
|  | Asian Spitz - European Mastiff | 0.578 | 0.601 | 0.962 | 126 | 0.916 | -1.029 | 2.185 |
|  | Retrievers - New World | -1.102 | 0.825 | -1.336 | 46 | 0.811 | -3.46 | 1.256 |
|  | Retrievers – European Mastiff | 0.392 | 0.78 | 0.503 | 36 | 0.952 | -1.577 | 2.362 |
|  | New World - European Mastiff | 1.495 | 0.776 | 1.926 | 56 | 0.457 | -0.767 | 3.756 |
| The sequential Sidak adjusted significance level is 0.05.  Confidence interval bounds are approximate. | | | | | | | | |

**Table S4. Pairwise comparison of** **the average change (Test1 – Test2) in “difficulty in Discrimination learning” score across breed clades.** Breed comparisons that are bolded had a statistically significant difference in their mean-level performance.

| Breed clade | | Mean Difference | Std. Error | Sig.^a^ | 95% Confidence Interval for Difference^a^ | |
| --- | --- | --- | --- | --- | --- | --- |
|  |  |  |  |  | Lower Bound | Upper Bound |
| UK Rural | Asian Spitz | 0.049 | 0.851 | 1 | -2.389 | 2.486 |
|  | Retrievers | -0.258 | 0.82 | 1 | -2.606 | 2.091 |
|  | New World | 0.607 | 0.84 | 0.998 | -1.799 | 3.012 |
|  | European Mastiff | -0.707 | 0.812 | 0.992 | -3.031 | 1.617 |
| Asian Spitz | UK Rural | -0.049 | 0.851 | 1 | -2.486 | 2.389 |
|  | Retrievers | -0.306 | 0.842 | 1 | -2.718 | 2.105 |
|  | New World | 0.558 | 0.862 | 0.999 | -1.909 | 3.024 |
|  | European Mastiff | -0.756 | 0.834 | 0.99 | -3.143 | 1.631 |
| Retrievers | UK Rural | 0.258 | 0.82 | 1 | -2.091 | 2.606 |
|  | Asian Spitz | 0.306 | 0.842 | 1 | -2.105 | 2.718 |
|  | New World | 0.864 | 0.831 | 0.972 | -1.514 | 3.243 |
|  | European Mastiff | -0.449 | 0.802 | 1 | -2.745 | 1.846 |
| New World | UK Rural | -0.607 | 0.84 | 0.998 | -3.012 | 1.799 |
|  | Asian Spitz | -0.558 | 0.862 | 0.999 | -3.024 | 1.909 |
|  | Retrievers | -0.864 | 0.831 | 0.972 | -3.243 | 1.514 |
|  | European Mastiff | -1.314 | 0.822 | 0.699 | -3.668 | 1.04 |
| European Mastiff | UK Rural | 0.707 | 0.812 | 0.992 | -1.617 | 3.031 |
|  | Asian Spitz | 0.756 | 0.834 | 0.99 | -1.631 | 3.143 |
|  | Retrievers | 0.449 | 0.802 | 1 | -1.846 | 2.745 |
|  | New World | 1.314 | 0.822 | 0.699 | -1.04 | 3.668 |
| Based on estimated marginal means  a. Adjustment for multiple comparisons: Sidak. | | | | | | |

**Table S5.** **Fixed effects table from the mixed model analysis predicting “difficulty in Reversal learning” score (N=105).** Ball/toy responsiveness is written as “BTR”, Food responsiveness is written as “FR”, and overall Impulsivity score written as “DIAS”. Statistically significant variables are bolded.

| Fixed Effects^a^ | F | df1 | df2 | Sig. |
| --- | --- | --- | --- | --- |
| Corrected Model | 3.764 | 16 | 126 | <0.001 |
| **Breed clade*** | **4.964** | **4** | **71** | **<0.001** |
| Age | 0.002 | 1 | 148 | 0.969 |
| Sex | 0.173 | 1 | 29 | 0.680 |
| Sport training | 0.012 | 1 | 94 | 0.912 |
| Hand-touch experience | 0.148 | 1 | 55 | 0.702 |
| BTR | 0.606 | 1 | 41 | 0.441 |
| FR | 0.118 | 1 | 66 | 0.732 |
| DIAS | 2.986 | 1 | 25 | 0.096 |
| **Time*** | **16.829** | **1** | **72** | **<0.001** |
| Breed clade * Time | 0.891 | 4 | 67 | 0.474 |
| Probability distribution: Normal | | |  |  |
| Link function: Identity^a^ | |  |  |  |
| a. Target: difficulty in Reversal learning score | | | |  |

**Table S6. Pairwise comparison of the average change (Test1 – Test2) in “difficulty in Reversal learning” score across breed clades.** Breed comparisons that are bolded had a statistically significant difference in their mean-level performance.

| Breed clade | | Mean Difference | Std. Error | Sig.^a^ | 95% Confidence Interval for Difference^a^ | |
| --- | --- | --- | --- | --- | --- | --- |
|  |  |  |  |  | Lower Bound | Upper Bound |
| UK Rural | Asian Spitz | 0.833 | 0.578 | 0.81 | -0.823 | 2.489 |
|  | Retrievers | 0.445 | 0.557 | 0.996 | -1.15 | 2.04 |
|  | New World | -0.064 | 0.571 | 1 | -1.697 | 1.57 |
|  | European Mastiff | -0.108 | 0.551 | 1 | -1.687 | 1.47 |
| Asian Spitz | UK Rural | -0.833 | 0.578 | 0.81 | -2.489 | 0.823 |
|  | Retrievers | -0.388 | 0.572 | 0.999 | -2.026 | 1.25 |
|  | New World | -0.897 | 0.585 | 0.748 | -2.572 | 0.779 |
|  | European Mastiff | -0.941 | 0.566 | 0.65 | -2.562 | 0.68 |
| Retrievers | UK Rural | -0.445 | 0.557 | 0.996 | -2.04 | 1.15 |
|  | Asian Spitz | 0.388 | 0.572 | 0.999 | -1.25 | 2.026 |
|  | New World | -0.509 | 0.564 | 0.99 | -2.124 | 1.107 |
|  | European Mastiff | -0.553 | 0.545 | 0.976 | -2.113 | 1.006 |
| New World | UK Rural | 0.064 | 0.571 | 1 | -1.57 | 1.697 |
|  | Asian Spitz | 0.897 | 0.585 | 0.748 | -0.779 | 2.572 |
|  | Retrievers | 0.509 | 0.564 | 0.99 | -1.107 | 2.124 |
|  | European Mastiff | -0.045 | 0.558 | 1 | -1.643 | 1.554 |
| European Mastiff | UK Rural | 0.108 | 0.551 | 1 | -1.47 | 1.687 |
|  | Asian Spitz | 0.941 | 0.566 | 0.65 | -0.68 | 2.562 |
|  | Retrievers | 0.553 | 0.545 | 0.976 | -1.006 | 2.113 |
|  | New World | 0.045 | 0.558 | 1 | -1.554 | 1.643 |
| Based on estimated marginal means  a. Adjustment for multiple comparisons: Sidak. | | | | | | |

**Table S7. Fixed effects table from the mixed model analysis predicting “Perseverance” score (N=105).** Ball/toy responsiveness is written as “BTR”, Food responsiveness is written as “FR”, and overall Impulsivity score written as “DIAS”. Statistically significant variables are bolded.

| Fixed Effects^a^ | F | df1 | df2 | Sig. |
| --- | --- | --- | --- | --- |
| Corrected Model | 2.303 | 16 | 120 | 0.006 |
| Breed clade | 2.164 | 4 | 53 | 0.086 |
| Age | 0.233 | 1 | 17 | 0.635 |
| Sex | 0.355 | 1 | 69 | 0.553 |
| Sport training | 0.276 | 1 | 188 | 0.600 |
| Hand-touch experience | 1.042 | 1 | 93 | 0.310 |
| BTR | 1.789 | 1 | 27 | 0.192 |
| FR | 1.286 | 1 | 19 | 0.271 |
| DIAS | 1.466 | 1 | 105 | 0.229 |
| Time | 0.486 | 1 | 51 | 0.489 |
| Breed clade * Time | 0.645 | 4 | 59 | 0.632 |
| Probability distribution: Normal | | |  |  |
| Link function: Identity^a^ | |  |  |  |
| a. Target: Perseverance score | | |  |  |

**Table S8. Pairwise comparisons of the Estimated Marginal Means for “Perseverance” score across Test 1 and Test 2 within the studied breed clades.** Breed clades that are bolded had a statistically significant difference in their mean-level performance.

|  | | | | | | | |
| --- | --- | --- | --- | --- | --- | --- | --- |
| Breed Group | Contrast Estimate (Test 1 - Test 2) | Std. Error | t | df | Adj. Sig. | 95% Confidence Interval | |
|  |  |  |  |  |  | Lower | Upper |
| UK Rural | -0.294 | 0.470 | -0.625 | 16 | 0.541 | -1.289 | 0.701 |
|  |  |  |  |  |  |  |  |
| Asian Spitz | 0.034 | 0.356 | -0.096 | 93 | 0.924 | -0.740 | 0.672 |
|  |  |  |  |  |  |  |  |
| Retrievers | 0.287 | 0.338 | 0.850 | 85 | 0.398 | -0.384 | 0.959 |
|  |  |  |  |  |  |  |  |
| New World | 0.148 | 0.269 | 0.548 | 193 | 0.584 | -0.384 | 0.679 |
|  |  |  |  |  |  |  |  |
| European Mastiff | 0.429 | 0.236 | 1.817 | 193 | 0.071 | -0.037 | 0.894 |
|  |  |  |  |  |  |  |  |
| The sequential Sidak adjusted significance level is 0.05. | | | | |  |  |  |
| Confidence interval bounds are approximate. | | | |  |  |  |  |

**Table S9. Pairwise comparison of the average change (Test1 – Test2) in “Perseverance” score across breed clades.** Breed comparisons that are bolded had a statistically significant difference in their mean-level performance.

| Breed clade | | Mean Difference | Std. Error | Sig.^a^ | 95% Confidence Interval for Difference^a^ | |
| --- | --- | --- | --- | --- | --- | --- |
|  |  |  |  |  | Lower Bound | Upper Bound |
| UK Rural | Asian Spitz | -0.26 | 0.508 | 1 | -1.713 | 1.193 |
|  | Retrievers | -0.581 | 0.489 | 0.934 | -1.981 | 0.819 |
|  | New World | -0.442 | 0.501 | 0.992 | -1.875 | 0.992 |
|  | European Mastiff | -0.723 | 0.484 | 0.774 | -2.108 | 0.662 |
| Asian Spitz | UK Rural | 0.26 | 0.508 | 1 | -1.193 | 1.713 |
|  | Retrievers | -0.321 | 0.502 | 0.999 | -1.759 | 1.116 |
|  | New World | -0.182 | 0.514 | 1 | -1.652 | 1.288 |
|  | European Mastiff | -0.463 | 0.497 | 0.987 | -1.886 | 0.96 |
| Retrievers | UK Rural | 0.581 | 0.489 | 0.934 | -0.819 | 1.981 |
|  | Asian Spitz | 0.321 | 0.502 | 0.999 | -1.116 | 1.759 |
|  | New World | 0.14 | 0.495 | 1 | -1.278 | 1.557 |
|  | European Mastiff | -0.142 | 0.478 | 1 | -1.51 | 1.227 |
| New World | UK Rural | 0.442 | 0.501 | 0.992 | -0.992 | 1.875 |
|  | Asian Spitz | 0.182 | 0.514 | 1 | -1.288 | 1.652 |
|  | Retrievers | -0.14 | 0.495 | 1 | -1.557 | 1.278 |
|  | European Mastiff | -0.281 | 0.49 | 1 | -1.684 | 1.122 |
| European Mastiff | UK Rural | 0.723 | 0.484 | 0.774 | -0.662 | 2.108 |
|  | Asian Spitz | 0.463 | 0.497 | 0.987 | -0.96 | 1.886 |
|  | Retrievers | 0.142 | 0.478 | 1 | -1.227 | 1.51 |
|  | New World | 0.281 | 0.49 | 1 | -1.122 | 1.684 |
| Based on estimated marginal means  a. Adjustment for multiple comparisons: Sidak. | | | | | | |

**Table S10. Fixed effects table from the mixed model analysis predicting “Emotionality” score (N=105).** Ball/toy responsiveness is written as “BTR”, Food responsiveness is written as “FR”, and overall Impulsivity score written as “DIAS”. Statistically significant variables are bolded.

| Fixed Effects^a^ | F | df1 | df2 | Sig. |
| --- | --- | --- | --- | --- |
| Corrected Model | 2.669 | 16 | 41 | 0.006 |
| Breed clade | 0.773 | 4 | 13 | 0.579 |
| Age | 1.030 | 1 | 2 | 0.415 |
| Sex | 0.180 | 1 | 13 | 0.679 |
| Sport training | 1.240 | 1 | 12 | 0.287 |
| Hand-touch experience | 0.865 | 1 | 12 | 0.371 |
| BTR | 0.925 | 1 | 36 | 0.343 |
| FR | 0.000 | 1 | 2 | 0.994 |
| DIAS | 592 | 1 | 49 | 0.445 |
| Time | 0.605 | 1 | 13 | 0.451 |
| Breed clade* Time | 1.891 | 4 | 8 | 0.208 |
| Probability distribution: Normal | | |  |  |
| Link function: Identity^a^ | |  |  |  |
| a. Target: Emotionality score | | |  |  |

**Table S11. Pairwise comparisons of the Estimated Marginal Means for “Emotionality” score between the studied breed clades within Test 1 and Test 2.** Breed comparisons that are bolded had a statistically significant difference between their mean-level performance.

| Time | Breed clade Pairwise Contrasts | Contrast Estimate | Std. Error | t | df | Adj. Sig. | 95% Confidence Interval | |
| --- | --- | --- | --- | --- | --- | --- | --- | --- |
|  |  |  |  |  |  |  | Lower | Upper |
| Test 1 | UK Rural – Asian Spitz | -0.176 | 0.283 | -0.623 | 21 | 0.993 | -1.009 | 0.656 |
|  | UK Rural - Retrievers | -0.069 | 0.321 | -0.215 | 45 | 0.996 | -0.87 | 0.733 |
|  | UK Rural – New World | -0.246 | 0.363 | -0.677 | 12 | 0.993 | -1.413 | 0.922 |
|  | UK Rural – European Mastiff | 0.077 | 0.266 | 0.289 | 41 | 0.996 | -0.611 | 0.765 |
|  | Asian Spitz - Retrievers | 0.108 | 0.273 | 0.394 | 193 | 0.996 | -0.595 | 0.81 |
|  | Asian Spitz - New World | -0.069 | 0.415 | -0.167 | 5 | 0.996 | -1.53 | 1.391 |
|  | Asian Spitz - European Mastiff | 0.253 | 0.207 | 1.224 | 82 | 0.921 | -0.342 | 0.849 |
|  | Retrievers - New World | -0.177 | 0.396 | -0.447 | 5 | 0.996 | -1.759 | 1.406 |
|  | Retrievers – European Mastiff | 0.146 | 0.181 | 0.808 | 193 | 0.993 | -0.359 | 0.651 |
|  | New World - European Mastiff | 0.323 | 0.38 | 0.85 | 5 | 0.993 | -1.419 | 2.064 |
| Test 2 | UK Rural – Asian Spitz | -0.378 | 0.796 | -0.475 | 4 | 0.962 | -3.472 | 2.715 |
|  | UK Rural - Retrievers | 0.503 | 0.561 | 0.898 | 60 | 0.962 | -1.054 | 2.061 |
|  | UK Rural – New World | -0.595 | 0.826 | -0.72 | 18 | 0.962 | -2.958 | 1.768 |
|  | UK Rural – European Mastiff | 0.28 | 0.576 | 0.487 | 89 | 0.962 | -1.143 | 1.703 |
|  | Asian Spitz - Retrievers | 0.881 | 0.633 | 1.392 | 4 | 0.913 | -2.595 | 4.358 |
|  | Asian Spitz - New World | -0.217 | 1.026 | -0.211 | 3 | 0.962 | -4.388 | 3.954 |
|  | Asian Spitz - European Mastiff | 0.658 | 0.647 | 1.017 | 3 | 0.962 | -2.975 | 4.291 |
|  | Retrievers - New World | -1.098 | 0.771 | -1.424 | 10 | 0.872 | -3.873 | 1.676 |
|  | Retrievers – European Mastiff | -0.223 | 0.34 | -0.656 | 193 | 0.962 | -1.094 | 0.648 |
|  | New World - European Mastiff | 0.875 | 0.799 | 1.096 | 13 | 0.938 | -1.732 | 3.482 |
| The sequential Sidak adjusted significance level is 0.05.  Confidence interval bounds are approximate. | | | | | | | | |

**Table S12. Pairwise comparison of the average change (Test1 – Test2) in “Emotionality” score across breed clades.** Breed comparisons that are bolded had a statistically significant difference between their mean-level performance.

| Breed clade | | Mean Difference | Std. Error | Sig.^a^ | 95% Confidence Interval for Difference^a^ | |
| --- | --- | --- | --- | --- | --- | --- |
|  |  |  |  |  | Lower Bound | Upper Bound |
| UK Rural | Asian Spitz | 0.202 | 0.508 | 1 | -1.254 | 1.657 |
|  | Retrievers | -0.572 | 0.49 | 0.94 | -1.975 | 0.83 |
|  | New World | 0.349 | 0.502 | 0.999 | -1.087 | 1.785 |
|  | European Mastiff | -0.203 | 0.485 | 1 | -1.591 | 1.185 |
| Asian Spitz | UK Rural | -0.202 | 0.508 | 1 | -1.657 | 1.254 |
|  | Retrievers | -0.774 | 0.503 | 0.743 | -2.214 | 0.666 |
|  | New World | 0.147 | 0.514 | 1 | -1.325 | 1.62 |
|  | European Mastiff | -0.405 | 0.498 | 0.996 | -1.83 | 1.02 |
| Retrievers | UK Rural | 0.572 | 0.49 | 0.94 | -0.83 | 1.975 |
|  | Asian Spitz | 0.774 | 0.503 | 0.743 | -0.666 | 2.214 |
|  | New World | 0.921 | 0.496 | 0.496 | -0.499 | 2.342 |
|  | European Mastiff | 0.369 | 0.479 | 0.997 | -1.002 | 1.74 |
| New World | UK Rural | -0.349 | 0.502 | 0.999 | -1.785 | 1.087 |
|  | Asian Spitz | -0.147 | 0.514 | 1 | -1.62 | 1.325 |
|  | Retrievers | -0.921 | 0.496 | 0.496 | -2.342 | 0.499 |
|  | European Mastiff | -0.552 | 0.491 | 0.953 | -1.958 | 0.853 |
| European Mastiff | UK Rural | 0.203 | 0.485 | 1 | -1.185 | 1.591 |
|  | Asian Spitz | 0.405 | 0.498 | 0.996 | -1.02 | 1.83 |
|  | Retrievers | -0.369 | 0.479 | 0.997 | -1.74 | 1.002 |
|  | New World | 0.552 | 0.491 | 0.953 | -0.853 | 1.958 |
| Based on estimated marginal means  a. Adjustment for multiple comparisons: Sidak. | | | | | | |


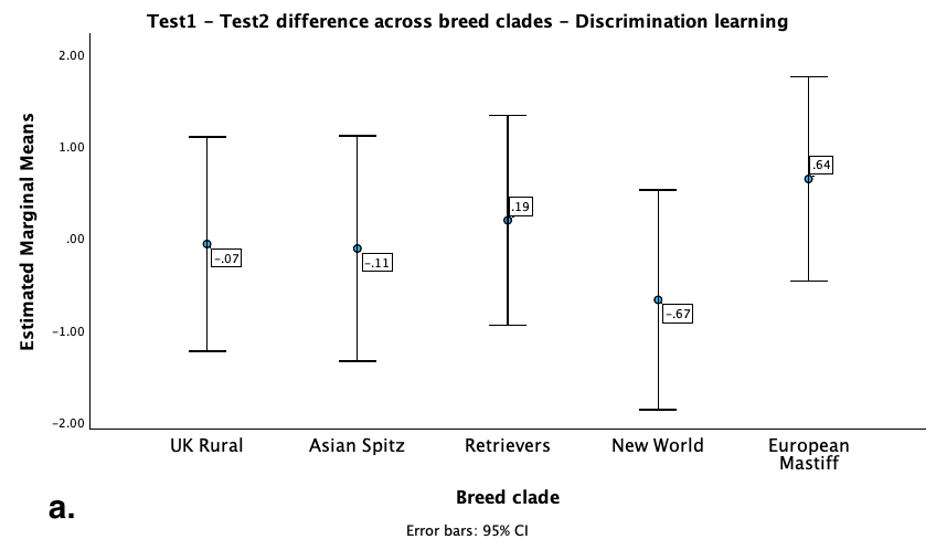


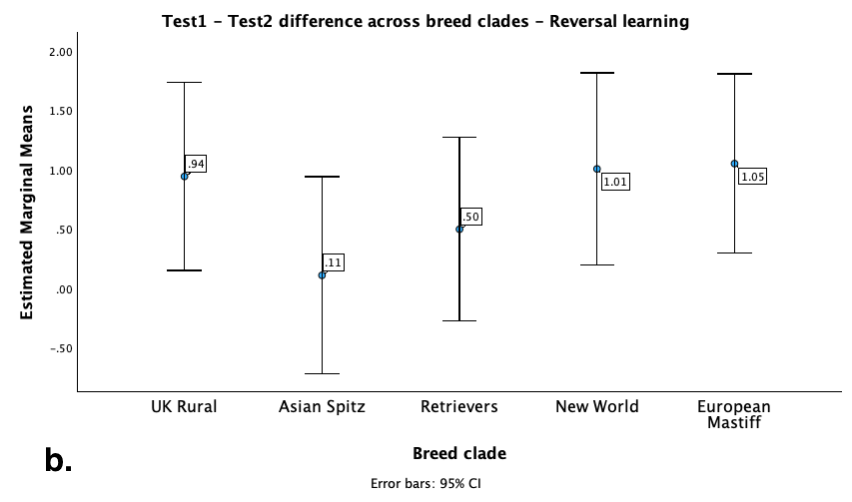


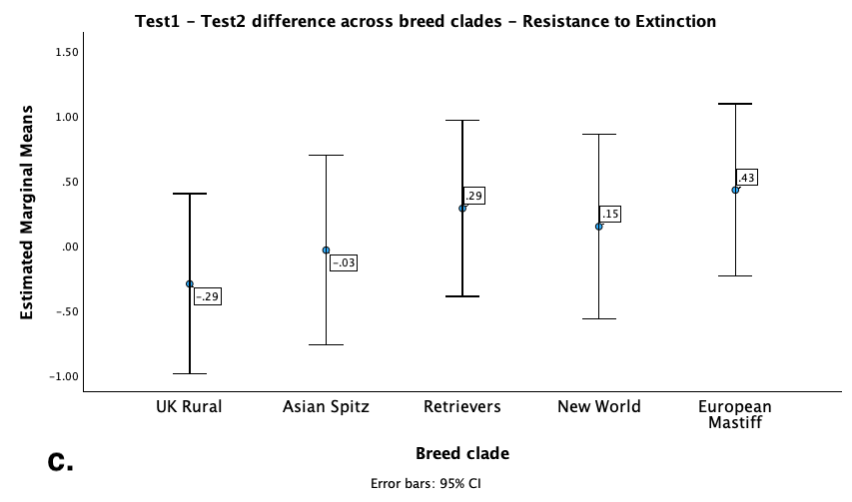


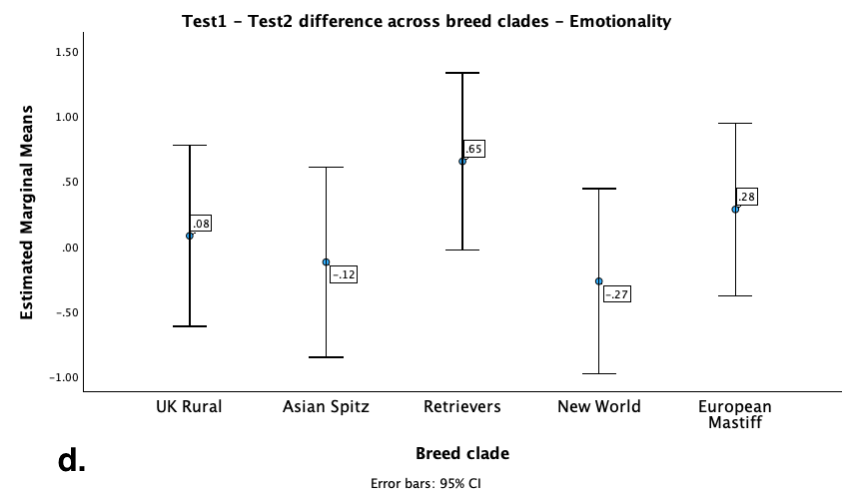


**Figure S1. The average (magnitude of) change (Test 1 – Test 2) in component scores: a) difficulty in Discrimination learning, b) difficulty in Reversal learning, c) Perseverance (resistance to Extinction), and d) Emotionality, across breed clades.** Higher scores describe more improvement in learning performance or higher levels of behavioural exhibition. Error bars are 95% Confidence Interval (CI) and values are the estimated marginal means.
